# Movement-Based Prosthesis Control with Angular Trajectory Is Getting Closer to Natural Arm Coordination

**DOI:** 10.3390/biomimetics9090532

**Published:** 2024-09-04

**Authors:** Effie Segas, Vincent Leconte, Emilie Doat, Daniel Cattaert, Aymar de Rugy

**Affiliations:** University of Bordeaux, CNRS, INCIA, UMR, 5287 Bordeaux, Franceemilie.doat@u-bordeaux.fr (E.D.);

**Keywords:** artificial neural network, human–robot interaction, movement-based, prosthesis control, trans-humeral limb deficiency

## Abstract

Traditional myoelectric controls of trans-humeral prostheses fail to provide intuitive coordination of the necessary degrees of freedom. We previously showed that by using artificial neural network predictions to reconstruct distal joints, based on the shoulder posture and movement goals (i.e., position and orientation of the targeted object), participants were able to position and orient an avatar hand to grasp objects with natural arm performances. However, this control involved rapid and unintended prosthesis movements at each modification of the movement goal, impractical for real-life scenarios. Here, we eliminate this abrupt change using novel methods based on an angular trajectory, determined from the speed of stump movement and the gap between the current and the ‘goal’ distal configurations. These new controls are tested offline and online (i.e., involving participants-in-the-loop) and compared to performances obtained with a natural control. Despite a slight increase in movement time, the new controls allowed twelve valid participants and six participants with trans-humeral limb loss to reach objects at various positions and orientations without prior training. Furthermore, no usability or workload degradation was perceived by participants with upper limb disabilities. The good performances achieved highlight the potential acceptability and effectiveness of those controls for our target population.

## 1. Introduction

Despite significant advances in the field of clinical arm prosthetics, particularly in improving hardware solutions and user interface designs, substantial challenges persist, especially in the area of control strategies proposed for individuals with severe disabilities, as is the case after trans-humeral amputation [1]. Indeed, for this level of disability, conventional control strategies based on myoelectric signals fail to intuitively coordinate and simultaneously control all lost degrees of freedom.

To enhance the intuitive nature of control, alternatives have been proposed using motor synergies, that is, repeatable coordination that naturally exists between the upper limb joints [2], to reconstruct the movements of the prosthesis. For instance, the remaining shoulder movements can be used to reconstruct the elbow flexion/extension [3,4,5] and, to a lesser extent, forearm pronation/supination [6,7]. However, these movement-based strategies have failed so far to reconstruct the wrist’s joints, which are nevertheless critical to correctly orient the hand to grasp objects [8].

On the other hand, the usefulness of computer vision algorithms has been demonstrated in studies using them to control the prosthesis hand configuration and opening width [9,10], as well as forearm pronation/supination [11,12], wrist flexion/extension [13], and radial/ulnar deviation [14]. Furthermore, significant advances in this field have enabled the location of objects to be grasped [15,16] and the determination of their position and orientation [17,18], along with the necessary hand configuration for grasping them [19].

Moreover, according to [20], motor synergies exist between the upper limb joints to maintain the task goal while allowing the necessary level of adaptability to accommodate unavoidable variations at individual joints. Therefore, in [21], we proposed merging these two approaches and demonstrated that adding information about the object’s position and orientation to a movement-based control helps reconstruct the degrees of freedom required to position and orient the hand so as to grasp that object. Following this study, we showed that tailoring our algorithm to the morphology of users, whether they have trans-humeral disabilities or not, enabled them to grasp objects placed in a wide range of positions and orientations, both in virtual reality and on a robotic setup, with movement times and performance levels comparable to that of natural movements [22].

As discussed in [22], however, an important limitation persists in this control. Although implementing a sort of natural inverse kinematic solving, thereby providing solutions representative of natural arm postures rather than merely optimizing for an arbitrary cost function, a discontinuity in distal angles occurs with this control at each change in movement goal. Indeed, as demonstrated in Supplementary Videos S1 and S2, the abrupt modification in the artificial neural network (ANN) inputs that occurs when a new movement goal appears simultaneously affects the ANN outputs. In a real-case scenario, such a discontinuity would lead to rapid and abrupt movements of the prosthesis joints, posing potential risks to the user, individuals in close proximity, and even the structural integrity of the prosthetic device itself.

To overcome this limitation, and to propose a control that eliminates this risk to the user, we introduce here an angular trajectory that smoothly transitions from the current distal arm configuration to a ‘goal’ configuration predicted by an ANN. The angular trajectory is based on a simple interpolation that takes into account the current gap between the current and the ‘goal’ configurations, the ongoing speed of the elbow (controlled exclusively by the user), and the remaining distance between the hand and the target.

Two methods, both relying on ANN predictions, are proposed to define the ‘goal’ configuration. The first method exclusively considers the information about the movement goal (i.e., the position and orientation of the target object relative to the user). However, this method assumes that the user will be able to correctly align its proximal joint congruently with the proposed distal posture and the current target. A potential limitation arises in scenarios where users are unable to or choose not to align the proximal joint accordingly, potentially hindering the target attainment. To counter this, we proposed a second method that incorporates both the target information and the proximal joint angles in the prediction of the ‘goal’ configuration. Indeed, according to the synergy theory proposed in [20], displacements of some joints adapt to compensate for the effect of other joints in order to maintain task completion despite unavoidable movement variability. Following this theory, the ‘goal’ distal configuration was adjusted in response to proximal movements in our second method in order to maintain task completion and cope with user variability in the control of its proximal joints.

In the present paper, we demonstrate that using these angular interpolation approaches to simulate the continuous control of a trans-humeral prosthesis enables naive participants, either valid (n = 12) or with a trans-humeral limb deficiency (n = 6), to transport a virtual hand to positions and orientations compatible with grasping objects spread across an extended working space. Notably, we demonstrated that participants with trans-humeral deficiency found this new approach to be as usable and undemanding as using their sound limb, highlighting their genuine interest in the approach and the significant potential for testing this control in real-life scenarios in future studies.

## 2. Methods

### 2.1. Participants

In this study, we utilized the Edinburgh handedness inventory (EHI, [23]) to assess valid participants’ handedness, with a score above 50 indicating right-handedness. We recruited twelve right-handed valid and six with trans-humeral limb deficiency participants (Age: 22 ± 2 and 49 ± 12 years old; EHI: 82 ± 17 and 5 with limb deficiency at the right side; six and five males, respectively). All participants were naive regarding the task, French speakers with normal or corrected-to-normal vision, and none of them had any mental or motor disorders that could potentially affect their task performance.

### 2.2. Experimental Setup

The participants were seated on a chair and wore a ViveTM Pro virtual reality headset (HTC Corporation). Up to four motion trackers (ViveTM Tracker, HTC Corporation, Taoyuan City, Taiwan) were secured using armbands to the participants’ trunk, upper arm, forearm, and hand, depending on the segments remaining. To prevent occlusions, four infrared beacons were strategically placed at each room’s corner. These beacons aided in reconstructing the 3D position and orientation of both the headset and the trackers within the virtual environment relative to a fixed reference frame. Data recording was performed at a 90 Hz sample rate thanks to SteamVR, Valve Corporation middleware. The Unity engine (Unity Technologies, San Francisco, CA, USA) managed the simulation of the virtual scene’s content and interaction with the participant, with a fixed refreshing rate of 90 Hz.

### 2.3. Virtual Arm Calibration and Kinematic Arm Model

**Figure 1:**
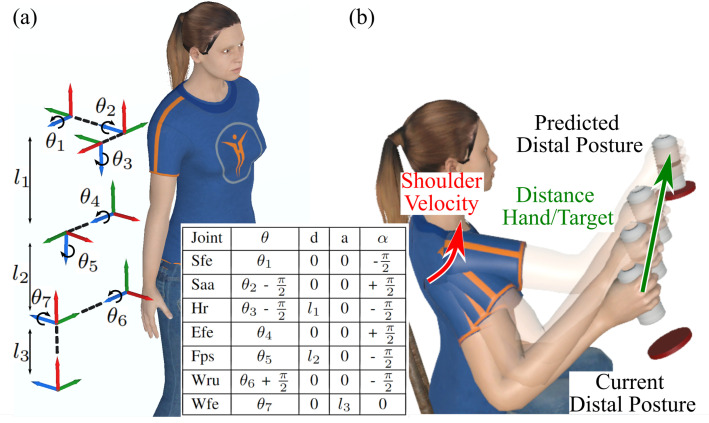
Methods. (**a**) 7 DoF kinematic arm chain and Denavit—Hartenberg parameters. (**b**) Control principle based on angular interpolation. The movement time is estimated from the speed of the hand that corresponds to the ongoing shoulder velocity, and the remaining distance between the hand and the target. The gap between the current and the ‘goal’ ANN-predicted distal posture is filled based on this estimated movement time.

After equipping the participant with virtual reality (VR) devices, a procedure was carried out to enable a virtual arm to replicate the movements of the participant’s actual arm. For a more detailed description of this procedure, readers may refer to the ’Virtual Arm Calibration’ section in [22]. At the conclusion of this procedure, the displacements of the trackers were linked to a seven degrees of freedom (DoF) virtual arm. This virtual arm was constructed by extracting seven anatomical arm angles: three for the shoulder (i.e., shoulder flexion/extension—Sfe, shoulder adduction/abduction—Saa, and humeral rotation—Hr), one for the elbow (i.e., elbow flexion/extension—Efe), one for the forearm (i.e., forearm pronation/supination—Fps), and two for the wrist (i.e., wrist radial/ulnar deviation—Wru and flexion/extension—Wfe), as per the kinematic arm model shown in Figure 1a. For the amputated participants, only the two first anatomical angles were extracted from the stump movements and linked to the virtual arm.

### 2.4. Ranges of Motion for Joint Angles

After the virtual arm calibration procedure was completed, the VR headset was temporarily removed to allow participants to observe and replicate basic movements for each DoF of their arm (Figure 1a), as demonstrated by an experimenter. Participants were instructed to execute these movements throughout the full range of motion of their joints so that the range of motion (ROM) could be estimated based on the extreme values achieved.

### 2.5. Task

In this study, participants were asked to pick up cylindrical objects and to place them on platforms positioned within their reachable workspace (see Supplementary Video S1). During the placement trials, a silhouette of the cylindrical object was displayed on the platform, acting as a visual guide to assist participants in understanding the intended final orientation and position of the object. The target location to reach was defined in terms of orientation along the sagittal and frontal axes, as well as the position of the middle point of either the physical object or the silhouette, depending on the type of trial (i.e., pick or place).

For a trial to be successful, participants had to maneuver the virtual hand in order to position it within a designated target validation zone, as defined by tolerance criteria related to both angle and distance relative to the target. These angle and distance values varied based on the experimental phase and were later referred to as ‘constraints’ in this article. Once the participant achieved this, the object turned red, signaling to the participant to press a keyboard button, thereby confirming the completion of the trial. Note that the participant is required to intentionally press a button to approximate prosthesis control, necessitating a conscious action (e.g., muscles co-contraction) to signal the intention to interact with an object to the system. Subsequently, a new trial commenced. Participants were subject to a predetermined time limit to complete a trial, the duration of which depended on the experimental phase. If this time limit was exceeded, the trial was deemed unsuccessful, and a new trial began. After every fifty trials, a pause was scheduled, providing participants a rest break. No data were recorded during these pause intervals.

Two sets of targets were generated for each valid participant. The first set, termed the ‘Exp1 Plausible Targets Set’, comprised 300 targets and was determined based on the participant’s joint angle ROM, with the elbow ROM reduced to 85% to prevent calculation errors caused by fully extended arm postures [22]. The second set, named the ‘Exp1 Possible Targets Set’, consisted of 200 targets and was derived from the actual arm movements performed by the participant during the initial acquisition phase. For further details on these target set generations, readers can refer to [22].

One set of targets, named ‘Exp2 Plausible Targets Set’, was generated for each amputated participant following the ‘Exp1 Plausible Targets Set’ generation procedure. It comprised 100 targets determined based on the participant’s shoulder joint angle ROM and the distal joint angle amplitude (5 to 95%) of the participants from [24] while performing a pick and place task.

### 2.6. Movement-Based Controls

This section delineates the arm control mechanisms employed in this study in comparison to the natural one (i.e., a seven DoF arm replicating the participant’s arm movements). Firstly, we introduce the artificial neural networks (ANNs) used in the experiment. Subsequently, we provide a concise review of the control methodology introduced in [22], complemented by an in-depth explanation of our novel control approach based on angular interpolation, which is proposed and tested in this study (Figure 1b).

#### 2.6.1. Artificial Neural Networks

The ANNs employed in this experiment were trained using natural movements executed during the initial acquisition phase by participants 1 to 12 from the database [24], a follow-up study that extends our previous database [22]. The training method described in [22] for the generic network was applied to adapt data from this database to match the user’s arm dimensions (see Figure 1 figure supplement 1b in [22]). In summary, the angular arm configurations performed by the database participants were applied to a virtual arm tailored to the user’s arm dimensions. For each sample, information about the orientation (i.e., frontal and sagittal angles) and position of a hypothetical target placed in the hand of the corresponding arm were computed (i.e., contextual information x, y, z, α, β in Table 1). Simultaneously, a collection of seven angles corresponding to the angular arm configuration was extracted (i.e., angular information Sfe, Saa, Hr, Efe, Fps, Wru, and Wfe in Table 1). All these variables were computed in a referential located at the shoulder position and oriented to match the trunk tracker orientation. These variables collectively constituted the training inputs and outputs of the diverse ANNs employed in this experiment (Table 1).

Similarly to the ANNs presented in [22], the **Proximo-Contextual (PC) ANNs** take as inputs the target’s position and orientation (i.e., frontal and sagittal angles) and the angular values of the two proximal joints (i.e., Sfe and Saa). The PC ANNs reconstruct the angular position of the five distal joints (i.e., Hr, Efe, Fps, Wru, and Wfe).

The **Contextual (C) ANNs** exclusively take the target position and orientation (i.e., frontal and sagittal angles) as inputs. They predict the seven angles of the kinematic arm model, although only the five distal ones were utilized in this study to reconstruct the virtual prosthesis configuration.

For the sake of comparison, a PC ANN, trained with the same learning rate and momentum as the generic ANN from [22], was employed in this experiment (Table 1, first line labeled PC−). These parameters were obtained due to a classical evaluation of the optimal training parameters (i.e., momentum and learning rate) for our neural networks with off-target and on-target errors taken into account to keep natural posture all along the hand transportation (i.e., from the initial pose to the target).

With our new approach, ANN predictions are not directly applied to the arm posture during hand transportation. Hence, only the on-target errors were considered relevant. Optimal training parameter evaluation indicated that better offline performances were achieved with a momentum of 0 and a learning rate of $10^{-4}$. Therefore, we introduced two new ANNs trained with these parameters, PC+ and C+ (Table 1, second and third lines). Both ANNs were used to predict the ‘goal’ distal configuration considered in the angular interpolation method (Figure 1b).

With the exception of the inputs and outputs (Table 1), all three types of ANNs share the same structure: two hidden layers, each comprising 256 neurons, one dropout layer set at 0.5, and one final hidden layer consisting of 64 neurons. All ANNs utilized in this study were trained using TensorFlow (1.14.0) with 10 epochs and a batch size of 128.

#### 2.6.2. Hybrid Arm Control

Our aim is to devise a prosthetic control strategy tailored for individuals with trans-humeral limb loss (i.e., to emulate the behavior of an upper arm stump fitted with a trans-humeral prosthesis). In [22], we introduced a movement-based approach that combined the natural movements of the user’s shoulder with the ANN’s predictions for the configuration of distal joints.

**Figure 2:**
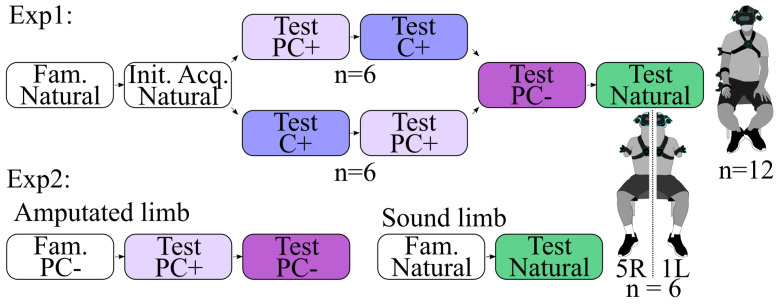
Protocol. Each box contains the phase name and the control used name, with Fam. and Init. Acq. standing for familiarization and initial acquisition phases, respectively. In Exp1, the order of the PC+ and C+ test phases was counterbalanced among participants. In Exp2, participants performed all the test phases with their stump except for the one with the natural control that they performed with their sound limb.

Consequently, at each time step, predictions from a PC− ANN were directly applied to the five distal joints of the virtual arm displayed in the virtual environment (i.e., Hr, Efe, Fps, Wru, and Wfe). Simultaneously, the two proximal joints (i.e., Sfe and Saa) remained under the direct control of the participant’s own shoulder movements. To enable a comparison with the results presented in [22], we replicated this control strategy, based on the predictions of a PC− ANN, in one of the phases of these experiments (Figure 2, Test PC−).

As discussed in [22], directly applying ANN predictions to the distal joints resulted in discontinuity when the target changed (i.e., an abrupt transition from one configuration to another in virtual reality; visible in Supplementary Video S2). In a real-world scenario, such discontinuity in the distal commands would lead to sudden and rapid adjustments in the prosthesis posture. These abrupt movements would pose inherent risks for the user and people in close proximity and may even compromise the structural integrity of the prosthetic apparatus itself. To address this concern, we propose the implementation of an angular interpolation method.

#### 2.6.3. Angular Interpolation Method

First, a ‘goal’ distal configuration is determined based on the ANN prediction, either PC+ or C+, depending on the control being tested. It is important to note that a physical prosthesis would be constrained from executing commands exceeding the ROM of its joints. In our effort to replicate the behavior of a physical prosthesis in this study, we imposed a saturation threshold on the ANN predictions. To emulate an ‘ideal’ prosthesis, the user’s maximal ROM was employed as a limit for each distal joint in the Exp1. In Exp2, the [22]’s Exp1 participants maximal ROM, calculated during the ROM for joint angle procedure, was used. Subsequently, the angular displacement yet to be achieved by each joint, $\Delta \theta_{i}\left(t\right)$, is determined by calculating the difference between the current angular value and the goal predicted value, as follows:

(1) $$\Delta \theta_{i}\left(t\right)=\theta pred_{i}\left(t\right)-\theta_{i}\left(t-1\right),$$

where $\theta pred_{i}\left(t\right)$ and $\theta_{i}\left(t-1\right)$ represent the ANN output and the angular value of the distal joint *i* at time *t* and t−1, respectively. If this difference is less than 1° for a distal joint, the ANN output is considered as reached and is directly provided as a command to the corresponding joint until a target modification occurs. However, if this difference exceeds 1°, an angular interpolation is executed to determine the new command to be sent to the distal joint.

For this purpose, the speed of the hand, $S_{h}\left(t\right)$, is approximated following

(2) $$S_{h}\left(t\right)=\frac{∥\vec{P_{hlock}\left(t\right)P_{hlock}\left(t-1\right)}∥}{T(t)-T(t-1)},$$

where T(t)−T(t−1) represents the elapsed time between *t* and t−1, and $P_{hlock}\left(t\right)$ and $P_{hlock}\left(t-1\right)$ represent, respectively, the hand positions at *t* and t−1 while the angular values of the five distal joints remain fixed. The hand’s position is evaluated with the distal joints kept fixed between t−1 and *t* to prevent the operation of a spurious feedback loop and ensure that changes in proximal joint angles are the sole factors influencing hand displacement.

If $S_{h}\left(t\right)>0$, the estimated movement time to complete the task, $\hat{M}T(t)$, is computed in

(3) $$\hat{M}T(t)=\frac{∥\vec{P_{target}\left(t\right)P_{h}\left(t\right)}∥}{S_{h}\left(t\right)},$$

where $∥\vec{P_{target}\left(t\right)P_{h}\left(t\right)}∥$ represents the remaining linear distance that the hand needs to travel to reach the target.

Finally, each angle displacement is calculated and added to the current angle value following:

(4) $$\theta_{i}\left(t\right)=\theta_{i}\left(t-1\right)+\frac{\Delta \theta_{i}\left(t\right)}{\hat{M}T(t)*Freq},$$

where Freq represents the frequency of 90 Hz at which the entire sequence, spanning from the ANN prediction to the angular displacement, is iterated to ensure the adaptation of the control to both the target location and the user’s movements.

It is noteworthy that in this experiment, Equation (4) can be reformulated as Equation (5), as the frequency at which data are received for the calculation is identical to the frequency at which the new command is applied.

(5) $$\theta_{i}\left(t\right)=\theta_{i}\left(t-1\right)+\Delta \theta_{i}\left(t\right)*\frac{∥\vec{P_{hlock}\left(t\right)P_{hlock}\left(t-1\right)}∥}{∥\vec{P_{target}\left(t\right)P_{h}\left(t\right)}∥},$$

This reformulation underscores the significance of hand displacement and the residual gap between the hand and the target.

### 2.7. Experimental Protocol

This section presents the successive phases of the experiment, as illustrated in Figure 2.

#### 2.7.1. Familiarization Phase

The familiarization phase is initiated at the outset of the experiment to acquaint participants with the apparatus, virtual environment, and experimental task. Thus, two familiarization phases are performed by the Exp2 participants, one with their stump (i.e., Exp2 first part) and one with their valid arm (i.e., Exp2 second part). During this phase, the displayed virtual arm emulates the movements of the participants’ arms in Exp1 and Exp2 second part or a hybrid arm with the PC− control in Exp2 first part. During the Exp1 and Exp2 second part familiarization phase, the participants are instructed to reach the initial targets from the Exp1 or Exp2 plausible target set, respectively. For Exp2 first part, they have to reach one time and a half the Exp2 possible target set to provide a familiarisation with the task itself comparable to the one of the Exp1 (Fam. and Init. Acq. phases). The trial validation constraints are set at 2 cm and 5° around the target in Exp1 to ensure maximum utilization of the ROM. In Exp2, they were set at 3 cm and 10° to accustom participants to the test phase’s constraints. A time limit of 5 s is imposed. Typically lasting less than 5 min in Exp1, the familiarization phase finishes once the participant can comfortably reach most targets.

#### 2.7.2. Initial Acquisition Phase

Following the familiarization phase, an initial acquisition phase was immediately conducted in Exp1. This phase shares similar characteristics with the familiarization phase, with the distinction that participants are instructed to reach all the targets within the Exp1 plausible target set. The arm configurations captured from participants during this phase are used to construct a customized Exp1 possible target set.

#### 2.7.3. Test Phases

Each participant completed one test phase per evaluated control (i.e., four in Exp1 and three in Exp2). In each test phase, the participants engaged in the pick-and-place task utilizing targets from either the Exp1 possible target set or the Exp2 plausible target set, depending on the experiment. In Exp1, validation constraints for the trials were set at 4 cm and 10° around the target, with a time limit of 10 s. In Exp2, they were set at 3 cm, 10° and 6 s.

In Exp1, the first two test phases were designed to compare the two movement-based controls with the angular interpolation method proposed in this article. To avoid any potential order effects, half of the participants started with an angular interpolation control based on predictions from a PC+ ANN, while the other half started with an angular interpolation control based on predictions from a C+ ANN. Afterward, two additional Test phases were implemented for comparative purposes. In the first one, participants controlled a hybrid arm where predictions from a PC− ANN were directly applied to the distal joints, similar to the approach detailed in [22]. In the second one, the virtual arm mimicked the natural arm movements of the participants.

In Exp2, due to the slightly better results obtained by Exp1’s participants with the PC+ compared to the C+ ANN, only the PC+ interpolation-based control was compared to the PC− ANN and the natural control with the sound limb.

### 2.8. Data Reduction and Metrics

Similar filtering procedures as outlined in [22] to eliminate significant motion capture measurement errors resulted in an average exclusion of 6.5 ± 8.1% and 7.6 ± 5.2% trials per participant and test phase in Exp1 and Exp2, respectively.

Subsequently, the study examined Exp1 and Exp2 participants’ ease of use based on performance and subjective metrics, as well as the characteristics of trajectories generated by the proposed controls in Exp1, both online and offline. The offline analysis aimed to study the control’s properties in a simulated scenario where participants were involved in reacting to the (possibly spurious) behavior of the control.

#### 2.8.1. Performance Metrics

The success rate (SR) is the percentage of trials in which participants pressed the validation button while the virtual hand was within the target validation zone before the allotted time expired. Given the consistently high SR achieved in all phases (all medians exceeding 92%), the subsequent performance and trajectory metrics were calculated exclusively on successful trials.

The movement time (MT) is defined as the duration between the appearance of a target and the moment the participant pressed the keyboard button to validate it.

The validation time (VT) measures the time elapsed from the last entry into the target validation zone to the moment the participant pressed the validation button. It serves as an indicator of the predictability of outcomes for the control methods under evaluation.

The shoulder position spread volume (SV) is determined by calculating the volume of the ellipsoid that should encompasses 97% of the shoulder positions recorded during a phase (see [21] for the computation method). It serves as an estimate of the compensatory movements involving trunk and shoulder translations.

With the exception of SV, which inherently produces a single value per phase, performance metrics are computed individually for each target.

#### 2.8.2. Subjective Metrics

After each test phase, the Exp1 participants were asked to complete two questionnaires assessing the perceived usability and workload of the control they used. To prevent any tracker displacement, the experimenter read and completed the questionnaire according to the participant’s answers. The headset was removed so that participants could also read the items. The participants were encouraged to request a repetition of a questionnaire item if needed.

The system usability scale (SUS) is employed to evaluate the perceived usability of each control [25]. The users rated their personal experience with the control using a 5-point Likert scale ranging from 1 (strongly disagree) to 5 (fully agree) for each of the 10 statements. The higher the computed score, ranging from 0 to 100, the more satisfactory the perceived usability was for the participant.

In this study, we used the French-validated version of the SUS [26], with wording adapted to our task. The scale proposed in [27] was utilized to assess the perceived usability level of the control being tested.

A homemade French translation of the Prosthesis Task Load indeX (Pros-TLX), an adaptation of the NASA-TLX for prosthesis use [28], was employed to evaluate the perceived workload. This questionnaire assesses eight dimensions known as limitations for prosthesis use (i.e., mental demands, physical demands, visual demands, conscious attention, frustration, situational stress, time pressure, and uncertainty). In the first part, users used a 20-point Likert scale ranging from 1 (low) to 20 (high) to express their agreement, regarding their experience with the control, with statements corresponding to each dimension. In the second part, users selected, from each of the 28 pairs of dimensions presented, the one that was most representative of their experience with the control. The higher the total score, ranging from 3.5 to 70, or a dimension score, ranging from 0 to 140, the greater the perceived workload, either overall or for that specific dimension.

#### 2.8.3. Online Trajectory Metrics

The spectral arc length (SAL), introduced in [29], quantifies the smoothness of virtual hand displacement. Ranging from −1 to −∞, this metric assesses the deviations from the mean movement velocity. A more negative SAL indicates greater deviation from the velocity mean, indicating a decrease in the smoothness of hand movement.

The curvature of the trajectory (CT), introduced in [30], evaluates the maximum distance of the hand from the straight line connecting the initial and final virtual hand positions (i.e., the minimum distance required to reach the target). A higher CT value suggests that the hand trajectory has deviated from the straight line, indicating a more curved trajectory.

The distance index (DI) measures the total distance traveled by the hand relative to the straight line as

(6) $$I_{dist}=\frac{\sum_{i=1}^{N}∥\vec{P_{h(t=i-1)}P_{h(t=i)}}∥}{∥\vec{P_{h(t=0)}P_{h(t=N)}}∥},$$

where $P_{h(t=x)}$ represents the virtual hand position at time x. The higher the DI value is, the more the distance traveled by the hand exceeds the straight line (i.e., shortest path).

#### 2.8.4. Offline Trajectory Metrics

In the offline analysis, each control strategy was applied to every successfully validated target during the TestNat phase of each Exp1 participant, utilizing the recorded shoulder movement.

The median mean absolute error (MMAE) is calculated as the median of the mean of the absolute differences between the actual and the predicted values for both proximal and distal joints, respectively.

The median hand position distance (MHPD) is determined as the median Euclidean distance between the position of the reconstructed hand and that of the actual hand.

The median hand orientation distance (MHOD) is defined as the median angle between the orientations of the reconstructed and actual hands. As no constraints were imposed on the rotational angle due to the cylindrical nature of the object, the angle is computed between the two vectors representing the rotational axes of the reconstructed and actual hands.

### 2.9. Statistical Analysis

In this experiment, a comparison was made between all the test phases (Nat, PC−, PC+, and C+ in Exp1 and Nat, PC−, and PC+ in Exp2) or the three tested movement-based controls (PC−, PC+, and C+). Metrics including MT, VT, SAL, CT, and DI were organized by participant and test phase, while MMAE, MHPD, and MHOD were organized by participant and movement-based control. Median values over multiple trials were extracted for each of these groupings. Notably, SR, SV, SUS scores, and Pros-TLX scores inherently provided a single value per participant and test phase. Consequently, we acquired datasets consisting of one value per participant for every combination of metric and control tested. First, the normality and variances homogeneity were assessed using Shapiro and Maulchly’s tests, respectively. Depending on the results, either a repeated measures ANOVA or a Friedman test was conducted followed, respectively, by Tukey or Conover post hoc tests if a significant difference was found at this level. The use of statistical tests based either on normality assumption or on the ranking procedure is prevented by the high success rates observed that led to equality between several participants. Thus, the statistical differences in success rate reported here were assessed by comparing the differences in the achievement of each target along all the phases of each experiment. First, a Cochran’s Q test for paired samples was performed, followed by a post hoc McNemar test if needed. Data processing and statistical analysis were carried out with the R software environment (version 4.1.1), with a significance threshold set at α = 0.05 with a Bonferroni correction applied if needed.

## 3. Results

### 3.1. Ease of Use Study

#### 3.1.1. Performance Metrics

**Figure 3:**
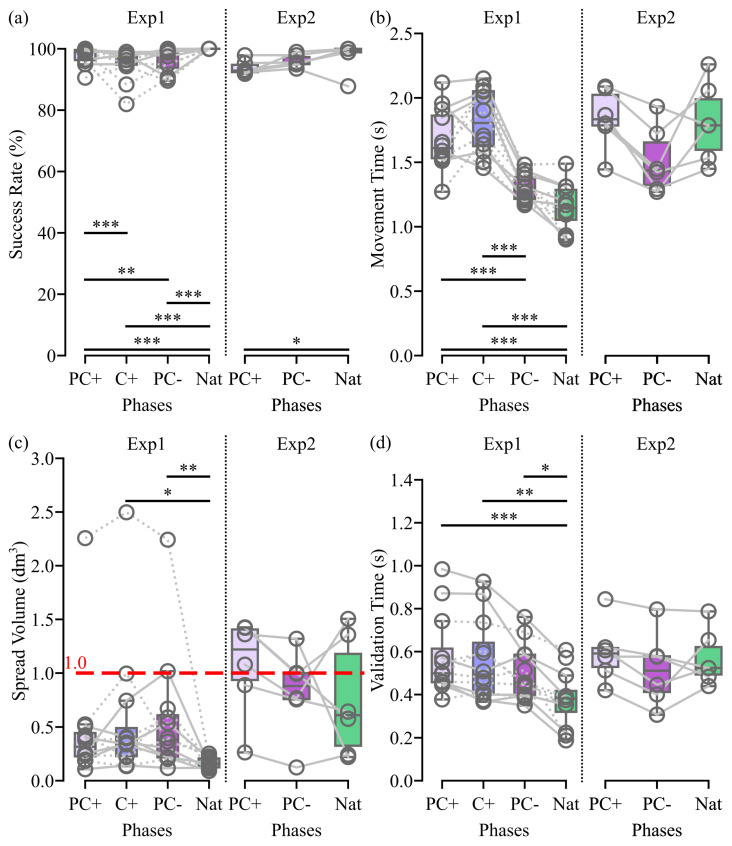
Exp1 and Exp2 Performance Metrics. The (**a**) success rate, (**b**) movement time, (**c**) shoulder spread volume, and (**d**) validation time are reported for each test phase. Each grey line corresponds to a participant with dashed and plain lines indicating participants who began by the control with the C+ and PC+ ANN, respectively. Box limits show the first and third quartiles, whereas the inside line shows the median value. Whiskers show min and max values without taking into account outliers. PC+, C+, PC−, and Nat represent the control used during the phases. Significant differences are indicated by stars, with * for *p* < 0.05, ** for *p* < 0.01, and *** for *p* < 0.001.

The high success rate observed in [22] remains consistent in this study, with all participants able to reach at least 81% of the presented targets, resulting in a median success rate exceeding 92% (Figure 3a). The proportion of validated targets is significantly higher when employing an angular interpolation control that considers the actual orientation of the shoulder (PC+) as compared to other control conditions in Exp1 or to the natural condition in Exp2.

However, in Exp1, the utilization of angular interpolation, regardless of whether shoulder orientation is considered or not, led to a significant increase in movement time compared to using either a PC− control or the natural condition (Figure 3b). Additionally, employing PC− or C+ control types resulted in significantly more shoulder movements than using the natural control (Figure 3c). Despite an increase in inter-individual variability, no significant difference was observed between the volume encompassed by the shoulder movements executed with a PC+ control and natural movements. Moreover, none of the medians exceeded 0.5 dm^3^. Furthermore, all three proposed controls were associated with a significant increase in the validation time (Figure 3d). This suggests a lack of sufficient predictability in our controls for naive participants, who were not always able to anticipate target attainment for their quick validation.

In Exp2, no significant differences were found between conditions for the movement time, shoulder spread volume, or validation time. This result might be due to the limited sample size coupled with the short exposure to the natural control for movement time and validation time or with the habit that amputated individuals have of making compensatory movements in their everyday lives for shoulder spread volume. However, despite a slight decrease in comparison with the Exp1 performances, these metrics indicate good performance level for Exp2 (i.e., close to 1 L for the shoulder spread volume and under 2 s for the movement time).

#### 3.1.2. Subjective Metrics

**Figure 4:**
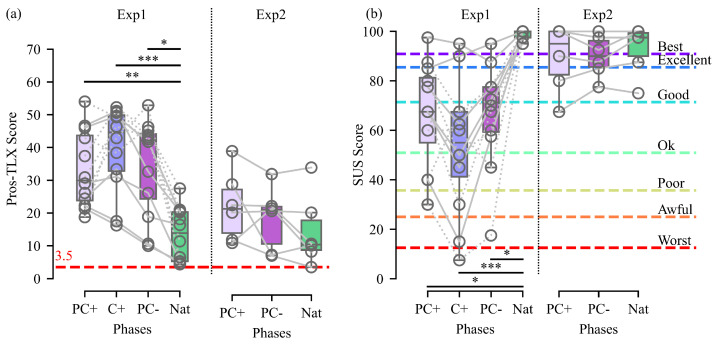
Exp1 and Exp2 Subjective Metrics. (**a**) Workload assessed with Pros-TLX scores and (**b**) usability assessed with SUS scores are presented for each test phase. Each grey line represents a participant, with dashed and solid lines indicating participants who began by the control with the C+ and PC+ ANN test phases, respectively. Box boundaries indicate the first and third quartiles, while the inner line represents the median value. Whiskers depict minimum and maximum values without taking into account outliers. PC+, C+, PC−, and Nat denote the controls used during the phases. Significant differences are indicated by stars, with * for *p* < 0.05, ** for *p* < 0.01, and *** for *p* < 0.001. The minimum possible Pros-TLX score is indicated by a line at 3.5. The colored dashed lines and corresponding adjectives are used to contextualize the SUS score associated with each control, according to [27].

The hypothesis that our controls might be insufficiently predictable for Exp1 participants is supported by a significantly higher workload (i.e., high Pros-TLX scores) and lower usability (i.e., low SUS scores) perceived by the participants while using our controls compared to the natural condition (Figure 4a,b, left sides, respectively).

The higher perceived workload is substantiated by the significantly greater uncertainty reported with our controls compared to the natural condition. Furthermore, the C+ and PC− controls led to a significantly increased reported level of frustration compared to the natural condition. Notably, this effect is not observed when using a PC+ control. However, the mean frustration reported for each of our controls is lower than that reported in [28] by participants without arm disabilities using a myoelectric-based control to open and close a prosthetic hand (Mean: TestPC+ = 13.3, TestC+ = 36.3, TestPC− = 30.4, TestNat = 1.8, calculated value in Figure 3 of [28] = 45.0). Nevertheless, due to the differences in protocols between these two experiments (i.e., virtual in this study versus physical in [28] and tasks involved), it is not possible to draw definitive conclusions without a direct comparison of the two approaches (i.e., our approach versus myoelectrical control), which should be performed in future studies.

Furthermore, despite the observed increase in the SUS score compared to the natural condition, the usability perceived by the participants while using our different controls could be qualified as ‘ok’ and is close to ‘good’ for the PC type ANNs, according to the scale proposed in [27].

Notably, no significant difference was found between the survey results obtained with our controls and the natural condition for participants with upper-limb disability in Exp2, indicating that they ranked our controls similarly well as a natural arm, both in terms of workload and usability (Figure 4a,b, right sides).

### 3.2. Trajectory Study

#### 3.2.1. Online Trajectory Metrics

As demonstrated in Supplementary Videos S3–S5, using angular interpolation methods allows for smooth transportation of the hand from one pose to another by smoothing out abrupt changes in the ANN predictions with each modification of the movement goal (see Supplementary Video S2 for an illustration of the problem).

**Figure 5:**
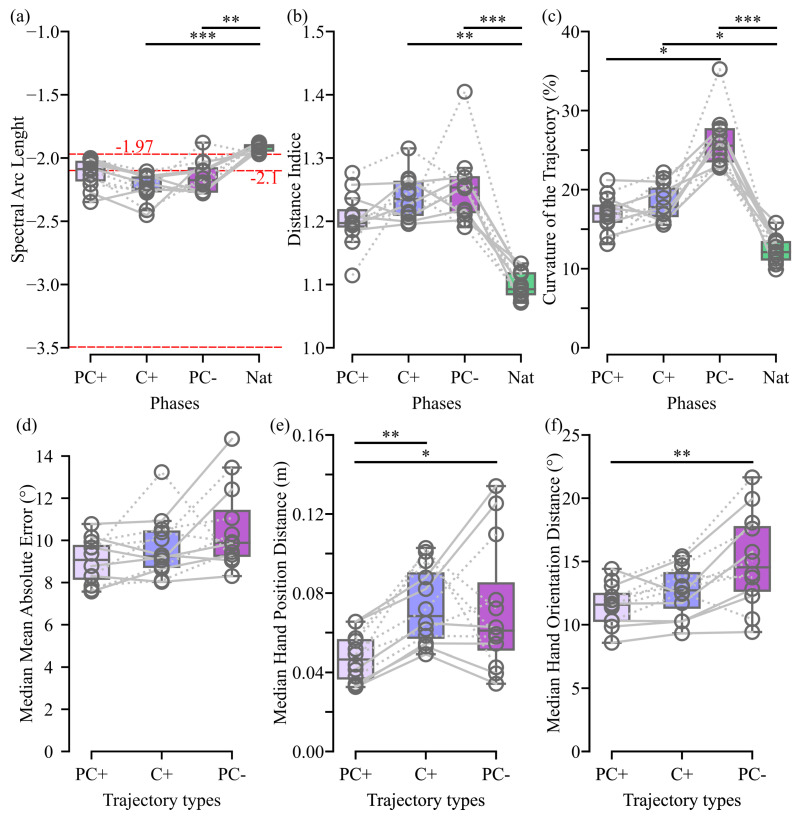
Exp1 Online and Offline Trajectory Study. (Upper line) The results for (**a**) the spectral arc length (SAL), (**b**) the distal index, and (**c**) the curvature of the trajectory are derived from data recorded during the experiment. PC+, C+, PC−, and Nat represent the controls used during the test phases. (Lower line) The results for (**d**) the median mean absolute error, (**e**) the median hand position distance, and (**f**) the median hand orientation distance are calculated based on simulated trajectories generated offline by the different control types. PC+, C+, and PC− represent the controls used to recreate the trajectories. Each grey line corresponds to a participant of the present study, with dashed and solid lines denoting participants who began with the C+ and PC+ ANN controls, respectively. Box plots display the first and third quartiles, with the inner line indicating the median value. Whiskers represent the minimum and maximum values without taking into account outliers. Red dotted lines in the SAL graph, with value sourced from [29], are included for comparison. The −2.1 and −1.97 lines indicate the SAL of a participant not suffering from any arm disability performing a 2D reaching task with a force field before and after the learning of the task, respectively. The −3.5 line represents the SAL of a hemiparetic patient performing the task after 30 rehabilitation sessions. Significant differences are denoted by stars, with * for *p* < 0.05, ** for *p* < 0.01, and *** for *p* < 0.001.

However, using the C+ and PC− controls resulted in significantly less smooth movements and longer distances traveled by the hand compared to those observed within the natural condition (Figure 5a,b). Nevertheless, the differences in fluidity found between the natural condition and our angular interpolation-based controls are smaller than those expected before and after learning of a 2D pointing task with a force field (differences: PC+ vs Nat = 0.2, C+ vs Nat = 0.3, before vs. after training [29] = 0.4). Furthermore, all medians are closer to the expected value after training than the value expected for a post-stroke hemiparetic patient learning a 2D pointing task (−1.9 and −3.5, respectively; [29]). All the controls proposed except PC+ led to a significantly curvier trajectory than the natural condition (Figure 5c).

However, despite no significant differences being found, the curvature of the trajectories elicited by the controls based on angular interpolation are closer to the natural condition ones than the ones for the PC− control. Moreover, in addition to the fact that trajectories generated using the PC+ control are not significantly curvier than those generated with the natural control, they exhibit a significant decrease in curvature compared to those generated with the PC− control.

#### 3.2.2. Offline Trajectory Metrics

**Figure 6:**
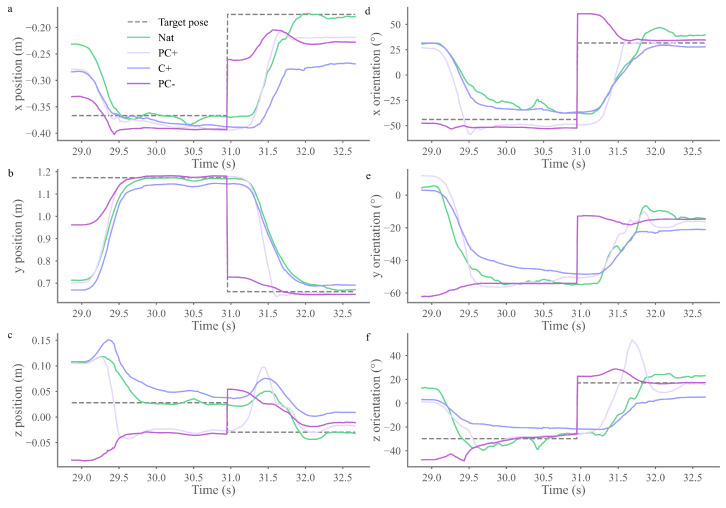
Exp1 Offline Trajectory Study. The hand’s position (**a**–**c**) and orientation (**d**–**f**) are depicted in the x, y, and z planes over time. The hand movement to reach two consecutive targets is illustrated, and the targets’ positions in x, y, and z, as well as their orientations in x and z, are shown in the corresponding graph for comparison. The targets’ y orientation is omitted as no constraints were applied to this dimension (i.e., the user can reach the target in every orientation around this axis).

In line with the online videos, Supplementary Video S6, which provides an egocentric point of view where each control is used as if the user had perfect upper-limb and trunk movements (i.e., without compensations), demonstrates the ability of the angular interpolation controls to mitigate changes in ANN predictions. Figure 6 provides a graphical view of the hand displacement in x, y, and z to reach two consecutive targets when each control is applied to a natural shoulder movement. It demonstrates how our new approach erases the discontinuity seen at each target change with PC− control by providing a smooth transition.

Furthermore, as can be easily grasped in the video, statistical differences emerged in the median hand position distance between the PC+ and the two other trajectory types and in the median hand orientation distance between the PC+ and the PC− trajectory type, with the PC+ trajectory type being closer to the natural trajectory (Figure 5e,f). Although no significant difference is observed, a similar trend was observed in the median mean absolute error (Figure 5d).

## 4. Discussion

The main goal of this study was to enhance the safety of our control by smoothing the transitions between targets and avoiding the sudden changes in prosthesis posture discussed in [22]. Here, we effectively eliminate the discontinuity in ANN outputs that arises from target changes by incorporating angular interpolation based on the remaining joint movements. Importantly, this interpolation method preserves the user’s capacity to accurately position and orient the virtual hand for grasping different targets (Figure 3a) without increasing the compensatory movements involved (Figure 3c) or degrading the usability and workload perceived (Figure 4), all while enabling a smooth transition from one arm configuration to the configuration that best aligns with the user’s intended aim (Supplementary Videos S3–S6). Despite the slight increase in the movement time observed (Figure 3b), we believe that these promising results pave the way for the use of this novel control scheme on a real prosthesis.

While the close-to-natural movement time reported in [22] is reproduced in the present study, the incorporation of angular interpolation into the control scheme has led to a moderate, yet significant, increase in the overall movement duration. This increase may be attributed, at least in part, to uncertainty regarding the control outcomes, which could also account for the extended validation time observed. In the control scheme proposed in [22], this effect may have been obscured by the abrupt change in the distal posture that occurred when a new target was presented. By instantaneously bringing the hand close to the target, it might have reduced the displacement and, consequently, the time required to reach the object. Nevertheless, the overall movement duration remains below 2 s, which is both way lower than (9 s in [31]), and comparable to (1.65 s for artificial and 1.3 s for natural control when transporting objects to the mouth in [32]) the results reported in the literature for virtual reality placement tasks. Furthermore, compared to our approach, this literature emphasized less the final position and orientation of the hand (only two postures in [31] and one hand position in [32]) and proposed to control fewer joints by natural shoulder movements (Efe and Fps in [31,32]). Moreover, it is worth noting that the results based on the utilization of interpolation methods presented herein were obtained from participants who were not provided any guidance regarding the control strategy and had not undergone any training specific to this particular control scheme. It could be argued that uncertainty, and thus the movement time, could decrease with the augmentation of the user expertise, as would be the case in a real-case scenario. Furthermore, in this study, we compared movements controlled by our novel control schemes to the gold standard of natural movements performed with a valid arm. Hence, when compared to the results in the relevant literature, the slight increase in movement duration appears quite minimal when considering the advantages offered by the proposed controls (e.g., directly usable by participants with trans-humeral limb deficiency, high success rate, complete reconstruction of all joints from the shoulder to the hand, accessibility to numerous positions and orientations without the need for training, and smooth transitions between postures).

It has been demonstrated that natural movements following a curvilinear trajectory, both in 2D [33] and 3D [34], are inherently longer in duration compared to an equivalent linear motion. Consequently, the curvier, and thus longer, trajectories obtained with interpolation methods may partly account for the observed increase in movement time. The curvature in these trajectories primarily stems from the limitations of our control scheme in generating the intricate angular movements executed by the joints required to maintain a near-linear displacement of the hand effector [35]. In the literature, several studies have focused on techniques designed to achieve such linear displacements [36,37]. However, these methodologies have typically failed to consider the curvier and more eccentric displacements observed in natural movements, as documented in [38]. Another approach involving the determination of predefined joint trajectories derived from natural movements performed in daily activities, which users can choose for task execution, has demonstrated its advantage over traditional myoelectric control methods [39]. Nevertheless, the available trajectories are constrained to those predefined within the system, far from encompassing the diversity of movements achievable with our upper arm. Future studies could leverage the two natural movement databases created by our team to explore alternative means of dynamically encapsulating the joint displacements of the human upper arm [24,40].

To define the ‘goal’ configuration for angular interpolation, we have introduced two methods, both relying on ANN predictions. The **first method** solely takes into account target information, such as its position and orientation relative to the user. However, it assumes that the placement of the proximal joint is congruent with the proposed distal posture for the current target. This assumption may be limiting in scenarios where users cannot or choose not to align the proximal joint accordingly, potentially hindering target attainment. To address this limitation, we have proposed a **second method** that incorporates both target information and proximal joint angles. This approach aligns with the concept of the compensatory synergies theories [20], in which the ‘goal’ distal configuration adjusts in response to proximal movements while maintaining task completion as the primary goal. Although not statistically significant in most of the metrics, this strategy tends to provide a better ease of use for the participants and improved trajectory metrics compared to the first approach.

Nevertheless, the performance of the method that solely considers the target information for movement planning is superior to the one achieved when relying solely on proximal joint displacements known by the ANN [21]. This demonstrates the importance of target information in determining distal joint angles. Therefore, the first approach remains highly valuable in situations where the precise proximal joint’s posture is not accessible.

Finally, it is important to stress that despite a slight degradation in performance, the functionality of our controls is still close to that of a natural arm, and that participants with upper-limb disabilities rated them much closer to a natural arm than valid participants did, both in terms of perceived workload and usability. Being confronted on a daily basis with control difficulties associated with their prosthesis, our amputated participants were probably much less affected by the slight degradation in performance and pleasantly surprised by the gain in functionality of our controls, which required no training.

### 4.1. Limitations

In the current study, our novel interpolation method facilitates a smooth approach toward the target (reaching) while preserving the participant’s ability to maintain control over their movement. However, before considering this new algorithm utilization for prosthesis manipulation, it is imperative to establish an efficient means of swiftly and accurately analyzing the visual scene observed by the user to obtain the aimed object pose. For this purpose, the use of camera-equipped glasses offers a viable solution to capture both the visual scene and the user’s gaze in an ecological set-up. Subsequently, computer vision techniques can be employed to determine the location and nature of the user’s target within a complex environment [15].

Although our approach brings the hand intuitively and smoothly in the vicinity of the target, queries persist regarding the subsequent phase: how to manage the grasping process, and what should be done once the object is grasped? To answer the first question, one could imagine complementing our approach by precise control of the hand degrees of freedom based either on myoelectric signals [31,41] or on computer vision techniques [9,10]. Regarding the latest concern, when the object is effectively grasped, it is possible to imagine a procedure to detect the targeted release placement based on the gaze direction [42] or to toggle change in the prosthesis mode based on biceps and triceps co-contraction [41]. Another limitation arises when the task performed is not a point-to-point task. As proposed above, a viable strategy could be to switch control methods or to use the gaze direction to determine the desired hand position. However, further study should determine suitable control for each specific task. However, it is worth noting that with a proper grasping process, our approach is sufficient to hold an object while the other hand interacts with it, facilitating bi-manual manipulation.

### 4.2. Future Work

In future studies, a direct comparison of our control scheme with other advanced control methods would be particularly valuable to demonstrate the respective advantages and limitations of each approach. For example, it could be interesting to propose that trans-humeral amputees assess the perceived usability and workload of our control scheme as an alternative to the control of a conventional myoelectric prosthesis with comparable degrees of freedom. Indeed, as demonstrated in [28], the perceived workload associated with a simple myoelectric control for opening and closing a prosthetic hand was reported to be worse than what we have observed, underscoring the potential advantages of our approach.

In this study, we utilized basic ANNs for their ability to uncover relationships between the different arm joints without the need for explicit specification. Coupling these ANNs with an angular interpolation method presents the advantage of a good trade-off between ease of use/deployment and effectiveness. However, a novel approach for regulating reaching movements could employ ANNs with the capability to directly manage trajectories, rather than depending on an interpolation method. For instance, long short-term memory (LSTM) algorithms have been used in the literature to determine the hand trajectory based on electroencephalogram signals [43] or to predict hip and knee angular trajectories based on the controlateral limb displacements [44]. Future studies should determine whether it is possible to reconstruct the arm distal joint displacements based on LSTM predictions.

After the abovementioned limitations have been addressed, the solutions assessed here in virtual reality would need to be tested on real settings, such as with the robotic platform Reachy [45] or its upgraded version presented during the Xprize avatar contest by Pollen Robotics [46], before being implemented on actual prosthetic devices.

Finally, given the interest shown by participants with trans-humeral disabilities, another potential application for this control scheme could be its use in the early stages of rehabilitation, as it does not require a mounted prosthesis.

## 5. Conclusions

Here, we demonstrated that a new prosthesis control based on angular interpolation enables naive participants, both valid or with trans-humeral limb disability, to reach, without any training, a variety of positions and orientations in virtual reality with close-to-natural movement times. By ensuring a smooth transition toward prosthetic joints predicted by the ANN involved, our new control strategy paves the way toward application in real-life settings.

## Figures and Tables

**Table 1:** ANN: inputs, outputs, and training parameters.

ANN	x	y	z	α	β	Sfe	Saa	Hr	Efe	Fps	Wru	Wfe	lr	mom.
PC−	*i*	*i*	*i*	*i*	*i*	*i*	*i*	*o*	*o*	*o*	*o*	*o*	1.59 × $10^{-7}$	0.95
PC+	*i*	*i*	*i*	*i*	*i*	*i*	*i*	*o*	*o*	*o*	*o*	*o*	$10^{-4}$	0
C+	*i*	*i*	*i*	*i*	*i*	*o*	*o*	*o*	*o*	*o*	*o*	*o*	$10^{-4}$	0

x, y, z = target position in x, y, and z, respectively; α, β= target orientation in the frontal and sagittal planes, respectively; Sfe, Saa, Hr, Efe, Fps, Wru, and Wfe = kinematic arm joints angular information; lr = learning rate; mom. = momentum; *i*= input; *o*= output.

## Data Availability

The dataset used to trained the ANNs is available online [24]. The code and dataset created and analyzed during this study are available from the corresponding author on reasonable request.

## Supplementary Materials

The following supporting information can be downloaded at: https://www.mdpi.com/article/10.3390/biomimetics9090532/s1, Video S1: A representative able-bodied participant from [22] performing the pick and place task. Video S2: Participant with trans-humeral disability from [22] performing the pick and place task with PC− control. Video S3: Valid participant performing the pick and place task with PC+ control. Video S4: Participant with trans-humeral disability performing the pick and place task with PC+ control. Video S5: Valid participant performing the pick and place task with C+ control. Video S6: Visualization of control behaviors if applied offline to movements performed naturally by a valid participant.

## Abbreviations

The following abbreviations are used in this manuscript:

ANN	Artificial neural network
PC	Proximo-contextual
C	Contextual
+/−	with or without angular interpolation
Nat	Natural
VR	Virtual reality
Sfe	shoulder flexion/extension
Saa	shoulder adduction/abduction
Hr	humeral rotation
Fps	forearm pronation/supination
Wru	wrist radial/ulnar deviation
Wfe	flexion/extension
LSTM	Long short-term memory
DoF	Degrees of freedom
EHI	Edinburgh handedness inventory
ROM	Range of motion
SR	Success rate
MT	Movement time
VT	Validation time
SV	Shoulder position spread volume
SUS	System usability scale
Pros-TLX	Prosthesis Task Load indeX
SAL	Spectral arc length
CT	Curvature of the trajectory
DI	Distance index
MMAE	Median mean absolute error
MHPD	Median hand position distance
MHOD	Median hand orientation distance.
