# Supplementary material for: Movement-Based Prosthesis Control with Angular Trajectory Is Getting Closer to Natural Arm Coordination
Source: Biomimetics (Basel). 2024 Sep 4;9(9):532. doi: 10.3390/biomimetics9090532 (PMC11430227; doi:10.3390/biomimetics9090532)
Supplement: Supplementary file 1 [file biomimetics-09-00532-s001.zip › biomimetics-3135767-supplementary.pdf]

Supplementary Materials

Video S1: A representative able-bodied participant from [22] performing the pick and place task. https://youtu.be/XPIlkrjwTtc
Video S2: Participant with transhumeral disability from [22] performing the pick and place task with PC- control. https://youtu.be/Utoa9aYWRK0
Video S3: Valid participant performing the pick and place task with PC+ control. https://youtu.be/eh4DrGggkak
Video S4: Participant with transhumeral disability performing the pick and place task with PC+ control. https://youtu.be/LCmUfG38Fa4
Video S5: Valid participant performing the pick and place task with C+ control. https://youtu.be/ziFIVXVKyyM
Video S6: Visualization of controls behaviors if applied offline to movements performed naturally by a valid participant. https://youtu.be/hGbOtOqiXeI
